# Changes in Circadian Rhythms Dysregulate Inflammation in Ageing: Focus on Leukocyte Trafficking

**DOI:** 10.3389/fimmu.2021.673405

**Published:** 2021-05-14

**Authors:** Poppy Nathan, Julie Elizabeth Gibbs, G. Ed Rainger, Myriam Chimen

**Affiliations:** ^1^ MRC-Versus Arthritis Centre for Musculoskeletal Ageing Research, Institute of Inflammation and Ageing, College of Medical and Dental Sciences, University of Birmingham, Birmingham, United Kingdom; ^2^ Centre for Biological Timing, Faculty of Biology Medicine and Health, University of Manchester, Manchester, United Kingdom; ^3^ Institute of Cardiovascular Sciences, College of Medical and Dental Sciences, University of Birmingham, Birmingham, United Kingdom

**Keywords:** inflammation, circadian rhythm, leukocytes, trafficking, chronotherapy

## Abstract

Leukocyte trafficking shows strong diurnal rhythmicity and is tightly regulated by circadian rhythms. As we age, leukocyte trafficking becomes dysregulated, contributing to the increased systemic, low-grade, chronic inflammation observed in older adults. Ageing is also associated with diminished circadian outputs and a dysregulation of the circadian rhythm. Despite this, there is little evidence to show the direct impact of age-associated dampening of circadian rhythms on the dysregulation of leukocyte trafficking. Here, we review the core mammalian circadian clock machinery and discuss the changes that occur in this biological system in ageing. In particular, we focus on the changes that occur to leukocyte trafficking rhythmicity with increasing age and consider how this impacts inflammation and the development of immune-mediated inflammatory disorders (IMIDs). We aim to encourage future ageing biology research to include a circadian approach in order to fully elucidate whether age-related circadian changes occur as a by-product of healthy ageing, or if they play a significant role in the development of IMIDs.

## Introduction

Under homeostatic conditions, leukocytes migrate between the vasculature and different tissues for immune surveillance. In response to infections or injuries, leukocytes are recruited to the site of inflammation and play key roles in pathogen clearance and tissue repair. Temporal expression of adhesion molecules on the leukocyte surface and on endothelial cells (ECs) mediates leukocyte trafficking, in a process known as the leukocyte adhesion cascade (1). Critically, it is essential that leukocyte trafficking is tightly regulated as aberrant leukocyte recruitment into tissues contributes to the development of most immune-mediated chronic inflammatory diseases (IMIDs).

Leukocyte trafficking was first identified to be under circadian control over 50 years ago, when it was observed that circulating lymphocyte numbers oscillate according to the time of day (2). Since then, core circadian machinery has been identified in almost all immune cells and a strong reciprocal relationship between immunity and circadian clocks has been well established (3–5). Disruption of circadian rhythms due to genetic manipulation or lifestyle (for example, shift work) dysregulates the immune response and increases the susceptibility to cancers, cardiovascular disease, and metabolic disease (6). Several IMIDs show daily patterns of symptom intensity and responsiveness to treatment which has created a new avenue of chronotherapy that involves optimal timing of drug delivery (4). In older adults, leukocyte trafficking becomes dysregulated, contributing to age-related low-grade, chronic inflammation (inflammageing) that predispose the older population to IMIDs (7). Although it is known there is a dampening of circadian rhythms with increasing age, little work has been done to investigate the effects of this circadian disruption on the increase in IMIDs observed in older adults.

In this review, we explore the current knowledge regarding circadian control of leukocyte trafficking and the circadian oscillations of inflammatory conditions. We discuss the changes that occur to the circadian clock with increasing age and investigate whether this may contribute the age-related increase in inflammation and diseases.

## Circadian Rhythm and Ageing

### The Master Pacemaker

The circadian rhythm refers to the endogenous cycles seen in nearly all organisms that correlate with the Earth’s 24-hour day-night cycle. Numerous biological processes are regulated by circadian clocks including behaviour, sleep, metabolism and body temperature (8, 9). In mammals, the master circadian pacemaker is found within the suprachiasmatic nucleus (SCN) which is entrained by the external environment and synchronises peripheral oscillators (10). Light enters the eye and sends input to the SCN *via* the retinohypothalamic tract enabling the central clock to entrain to external light/dark cues (11). SCN neurons send rhythmic outputs to peripheral organs and other brain areas, allowing global synchronisation with the external environment (12). Additionally, SCN neurons are able to generate autonomous circadian outputs allowing for circadian rhythms to exist even under constant darkness (13).

### Molecular Mechanisms

In mammalian cells, the intracellular circadian clock is made up of an autoregulatory negative feedback loop. Transcriptional activators CLOCK (Circadian Locomotor Output Cycles Kaput) and BMAL1 (Brain and Muscle ARNT-Like 1) dimerise and form a complex. The CLOCK/BMAL1 complex then translocates to the nucleus where they bind to E-Box elements in promoter sequences of clock-controlled genes to positively regulate their own transcription (14). CLOCK/BMAL1 also promotes transcription of the clock regulators, Cryptochrome (CRY) and Period (PER), which in turn dimerise and undergo nuclear translocation where they inhibit CLOCK/BMAL1, repressing their own transcription (15). In addition to this feedback loop of core clock genes, nuclear receptor subfamilies Rev-erb and ROR (retinoic acid-related orphan receptor) compete for binding to ROR responsive elements (ROREs) in *Bmal1* promoter sequences to repress and promote expression of BMAL1, respectively (16). Post-translational modifications of clock transcription factors further regulate this feedback loop. Phosphorylation of PER and CRY proteins by casein kinase Iϵ/δ and AMP kinase promotes ubiquitination by E3 ligases resulting in their degradation (8). Recently, another circadian repressor gene has been identified which is under control of the circadian clock (17). CHRONO (ChIP-derived repressor of network oscillator) inhibits CLOCK/BMAL1 transcription activation in a histone deacetylase (HDAC)–dependent manner, adding an epigenetic arm to the mammalian circadian clock (18). Overall, this negative feedback loop takes about 24 hours and results in the circadian oscillation seen in a multitude of physiological processes.

### Ageing and the Circadian Rhythm

It is well-established that the circadian system influences ageing and longevity, and vice versa. Circadian outputs are diminished in older animals (19); transplantation of foetal SCN tissue into aged hamsters led to increases in longevity and restored the age-associated loss of behavioural rhythmicity seen in control animals (20). BMAL1 knockout mice (KO) have significantly shorter lifespans than wild type (WT) controls, and display a premature ageing phenotype (21). Reactive oxygen species (ROS) accumulate in the kidney, spleen and heart of BMAL1 KO animals which all show an age-related decrease in size, suggesting a role of oxidative damage in age-associated degeneration. Inhibition of endogenous BMAL1 by siRNAs in murine fibroblast cell lines also increases ROS levels (6). Oxidative damage caused by increasing ROS production could drive the progression of cellular senescence of local cells, promoting a senescence-associated secretory phenotype, and subsequent dysregulation of the inflammatory response. CLOCK KO mice also have significantly reduced lifespans than WT controls, but show a milder ageing phenotype than BMAL1 KO mice, whereas PERIOD-deficient mice only have reduced lifespans after challenge with irradiation (22). The severe ageing phenotype limited to BMAL1 KO mice may be due to systemic effects independent of the circadian role of BMAL1, or functional redundancy seen by other core clock proteins. Importantly, BMAL1 KO mice also lose all time-of-day dependent leukocyte trafficking when housed in constant darkness, in contrast to WT littermates (23) indicating the pivotal role of the circadian clock in regulation of leukocyte trafficking.

In humans, ageing is associated with a reduced sleep quality and disrupted sleep cycles (9), which in turn further dysregulates the robustness of the circadian rhythm. Importantly, circadian rhythm dysregulation is associated with the development of age-related disorders, including inflammatory and metabolic disorders, and neurodegenerative diseases such as Alzheimer’s Disease and Parkinson’s (24). Several changes occur to the circadian clock with increasing age and identifying which of these are natural processes of healthy ageing and which of these are pathological will increase our understanding of age-associated aberrant inflammation.

## Circadian Regulation of Leukocyte Trafficking in Ageing

It is well established that leukocyte trafficking follows a circadian oscillation [reviewed extensively in (3, 4, 25)]. The expression of circadian clock genes is ubiquitous to nearly all immune cells, and clocks can directly regulate immune cell trafficking. Leukocyte trafficking becomes dysregulated in ageing as expression of adhesion molecules, chemokines and integrins change and senescent cells accumulate (26), contributing to inflammageing and increasing susceptibility to IMIDs. Despite the abundant research focusing on circadian changes with increasing age, very little work has been done on how this affects circadian control of leukocyte trafficking. Future research should concentrate on this interaction to identify which changes to the immune system and to the circadian machinery occur as a natural by-product of ageing and which are signs of pathology.

### Neutrophils

Neutrophils are the first innate immune cell recruited to sites of inflammation, where they phagocytose pathogens and secrete anti-microbial agents (27). Under steady state conditions, neutrophils are retained in the bone marrow by the key retention signal, CXCL12, acting through its receptor, CXCR4. Diurnal adrenergic signals inhibit CXCL12 expression in the bone marrow, resulting in daily variations in chemokine expression which regulates the circadian egress of neutrophils (and haematopoietic stem cells) from the bone marrow (28, 29). Following LPS challenge, neutrophils show a circadian-regulated recruitment to the lungs (30). Interestingly, this is regulated by the circadian clocks in lung epithelial cells, and not the clocks within neutrophils themselves. Local lung epithelial cells regulate the diurnal expression of the chemokine, CXCL5, in a glucocorticoid-dependent mechanism, which attracts neutrophils to the lungs (30). This identifies the complexity of circadian regulation of leukocyte trafficking, as immune cells both contain intrinsic clock machinery and are regulated by chemokine expression, which can be under the control of circadian clocks in other cells.

Recently, it has been reported that human and murine neutrophils possess an intrinsic, cell-autonomous diurnal ageing programme that acts to regulate trafficking of neutrophils to infections, whilst promoting their removal from the bloodstream, thus protecting vessels from inflammation (31). In young (6-12 weeks old) WT mice, neutrophils lose CD62L expression and gain CXCR4 as they age, promoting their recruitment to the bone marrow for elimination (32). This diurnal change is mediated by BMAL1, which upregulates expression of CXCL2, enables autocrine surface CXCL2-CXCR2 interactions and in turn promotes CD62L expression (31). Neutrophil-specific CXCR4 KO mice showed constitutive ageing as seen by low levels of CD62L (31). Persistence of aged neutrophils in the vasculature of these mice increased thrombo-inflammation in a model of ischemia-reperfusion, and depletion of these neutrophils prevented thrombus formation and improved survival after infarction (31). This suggests that the importance of this diurnal neutrophil ageing process is to prevent senescent neutrophils accumulating in the vasculature and to prevent thrombo-inflammation. Healthy aged mice have an accumulation of CD11b^high^/ICAM-1^high^ neutrophils in lymphoid organs (33) which may be in response to the increased levels of inflammation in aged mice. However, no research has been done on the diurnal neutrophil ageing process in aged mice and future work should aim to identify if this accumulation of neutrophils in aged lymphoid organs is a result of dysregulated neutrophil ageing due to reduced circadian outputs.

### Monocytes/Macrophages

Macrophages are key regulators of the innate immune response and show strong circadian oscillations in genes involved in cytokine secretion, which are essential mediators of leukocyte trafficking (34). REV-ERBα has been highlighted as a direct link between the circadian clock and the macrophage inflammatory response (35) prompting further investigation of this nuclear receptor as a therapeutic target. The role of REV-ERBα in immune responses is well established [reviewed in (36)], and recently synthetic REV-ERBα agonists are being used *in vivo* to investigate a direct circadian modulation in IMIDs such as in Rheumatoid Arthritis (RA) and colitis. RA shows strong symptom rhythmicity, underpinned by daily fluctuations in serum IL-6 concentrations (37). Synthetic REV-ERB ligands have been shown to control the release of IL-6 from macrophages and can alleviate disease symptoms (38). REV-ERBa also has a protective effect against colitis *via* down-regulation of Nlrp3 inflammasome activity (39). Activation of REV-ERBα ameliorates ulcerative colitis in WT mice (39) suggesting it may be a promising target for colitis treatment.

Several aged-related changes occur in macrophages, including polarization towards an alternate M2 phenotype and reduction in phagocytosis (40). Despite M2 macrophages originally considered to display an ‘anti-inflammatory’ phenotype, these age-associated M2 like macrophages secrete several pro-inflammatory mediators including TNFα, IL-1 and IL-6. In mice, Ly6C^high^ (inflammatory) monocytes but not Ly6C^low^ (patrolling) monocytes exhibit diurnal oscillations in trafficking under both homeostatic conditions and in a model of sterile peritonitis (41). A recent study discovered BMAL1 is induced following stimulation of M1, but not M2, macrophages by inflammatory stimuli (42). It appears that the classically activated, pro-inflammatory ‘M1’ phenotype is more tightly regulated by the circadian clock. Very little research has been done on the effect of ageing-associated circadian dysregulation and macrophage function, and it would be interesting to understand how the clock alters within ageing macrophages and whether circadian control of transcriptional programmes is affected. As discussed above, it appears that circadian regulation can have varying effects on different macrophage subsets, which highlights the complexity of the relationship between inflammation and circadian clocks. Targeting circadian mechanisms may be important to maintain homeostasis and responses to inflammation.

### Lymphocytes

Numbers of T-cells in the circulation follow daily oscillations, with the highest numbers during the behavioural rest phase and decreasing up to 40% at the peak of the active phase (43). However, individual T-cell subsets show varying migration patterns throughout the day, which is regulated by varying changes in serum concentrations of glucocorticoids and catecholamines (43). Cortisol levels peak in the blood at the beginning of the active phase and up-regulates IL-7 receptor (IL-7R) and the chemokine receptor, CXCR4, on the surface of naïve and central memory T-cells, mediating their extravasation into the bone marrow. Conversely, numbers of circulating effector CD8^+^ T-cells peak during the active phase at the same time as epinephrine. Effector CD8^+^ T-cells have increased intrinsic expression of beta-2-adrenergic receptors and CXCR1, which is proposed to be the reason for the effector CD8^+^ T-cell response to epinephrine. This subset-specific variation in trafficking is thought to provide increased immune defence during the active phase, when injury or infection is most likely to occur (43, 44).

Numbers of lymphocytes in the lymph nodes fluctuate in the opposite manner than those in the circulation. In young WT mice, migration of lymphocytes to lymph nodes peaked at the start of the active phase roughly 8 hours after peak blood lymphocyte concentrations (45, 46). BMAL1 regulates rhythmic expression of CCR7 and the sphingosine-1-phosphate receptor (SIPR1) on lymphocytes, which mediates their homing to and egress from lymph nodes respectively (45). Lymphocyte egress from lymph nodes is also regulated by adrenergic innervation through B2-adrenergic receptors (AR) (46). B2-AR-deficient mice lost the daily fluctuations of lymphocyte numbers in blood and lymph nodes due to reduced levels of norepinephrine in peripheral lymph nodes (46). It is thought that circadian oscillations in immune cells prime the immune system for stronger responses in the active phase when interaction with pathogens are most likely to occur. Retention of lymphocytes within the lymph nodes during the active phase is thought to increase the chance to encounter T-cells with their cognate antigen. These studies highlight the importance of both cell-intrinsic clocks and cell-extrinsic rhythmic signals for driving daily trafficking of lymphocytes.

T-cell recruitment is impaired in older adults, leading to a compromised adaptive immune response, increased vulnerability to infections, and weakened responses to vaccinations (reviewed in (26). Age-related dysregulation of lymphocyte recruitment has mostly been attributed to changes in expression of chemokines and adhesion molecules, and accumulation of senescent immune cells. However, very little research has investigated the contribution of diminished circadian outputs on lymphocyte trafficking in ageing.

### Endothelial Cells

**Figure 1:**
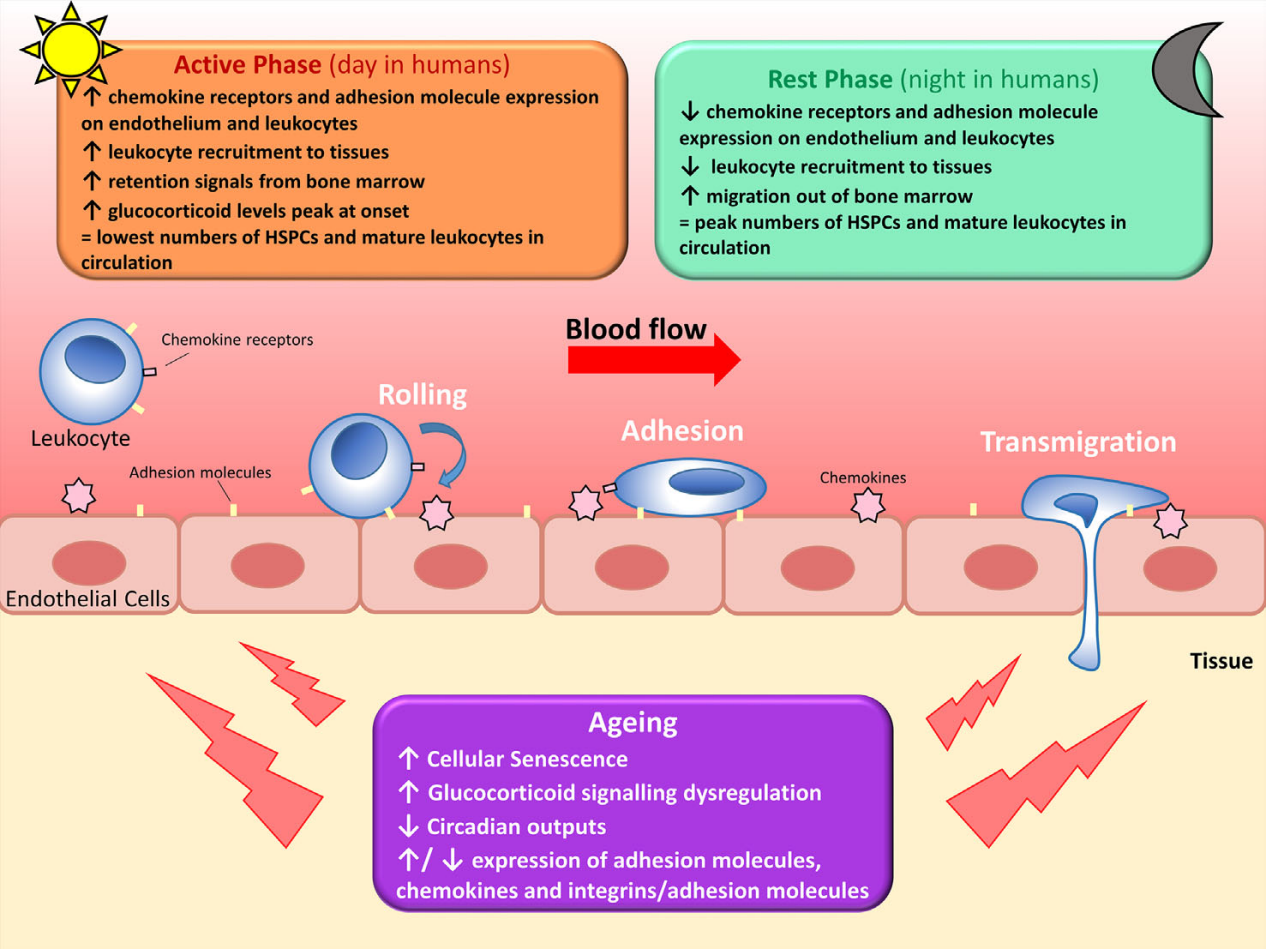
Circadian regulation of leukocyte trafficking. Migration of leukocytes such as lymphocytes, neutrophils and monocytes, out of circulation and into surrounding tissues is regulated by circadian clocks. Hematopoietic stem cells (HSPCs) and mature leukocytes (except CD8^+^ T cells) peak in the circulation during the rest phase as there’s less migration out of the blood and increased migration of leukocytes and haematopoietic stem cells out of the bone marrow. Conversely, circulating HPSCs and mature leukocytes (except CD8^+^ T cells) are at their lowest during the active phase due to increased leukocyte recruitment to tissues and reduced migration out of the bone marrow. Leukocyte migration is regulated by diurnal changes in expression of chemokines and adhesion molecules, and fluctuating glucocorticoid levels and adrenergic signalling. With increasing age, leukocyte trafficking becomes dysregulated due to a multitude of factors. Senescent cells accumulate and increase secretion of pro-inflammatory cytokines, glucocorticoid levels decrease and signalling becomes dysregulated, several age-associated changes occur to cytokine and adhesion molecule expression, and circadian outputs diminish. All of these contribute to dysregulated leukocyte trafficking seen in older adults.

Leukocyte recruitment is also regulated by oscillating expression of adhesion molecules on the EC surface. Autonomic innervation *via* β-adrenoreceptors differentially regulates adhesion molecule expression in different tissues (23), resulting in a highly-tissue specific temporal expression of EC adhesion molecules. A screen of adhesion molecule expression in multiple murine organs revealed a general peak in expression of adhesion molecules on ECs at the start of the active phase, parallel to the increased leukocyte emigration from blood (47). Adoptive transfer of cells into EC-specific Bmal1-deficient mice lost the time-of-day dependent leukocyte migration out of the circulation seen in WT controls (47). Therefore, leukocyte recruitment is regulated by rhythmic expression of adhesion molecules on both the EC surface and the leukocyte surface, increasing efficacy of the leukocyte-endothelium interaction required for leukocyte rolling, adhesion, and transmigration across the endothelial barrier (**Figure 1**).

## Circadian Rhythms and the Ageing Immune Response

### Vaccination

Older adults (>65 years) often have weaker responses to primary vaccination than younger adults, in terms of titre and immunity to infection (48, 49). Older adults are particularly susceptible to infections and are at increased risk for serious complications due to ageing-related comorbidities and increased immunosenescence (50). Therefore, vaccine optimisation is essential to limit hospitalisation and deaths due to vaccine-preventable infections in the older population. Interestingly, Suzuki et al. (46) showed that in young (8-12 week) WT mice immunisation *via* intradermal injection of a soluble antigen conjugated with chicken γ-globulin (NP-CGG) resulted in an elevated humoral response when administered at peak lymph node lymphocyte cellularity (46). Recently, this has been confirmed in humans by administration of BCG vaccines. Early morning vaccination produced a stronger adaptive immune phenotype and increased cytokine production compared with later morning and evening administration (51). The immune microenvironment present during the initiation of an adaptive response is therefore an important regulator of the overall strength of the response, and timing of vaccine administration needs to be considered when developing and researching novel vaccines (52).

### Circadian Misalignment

The importance of a robust circadian rhythm for maintaining health span with increasing age is evident as chronic circadian misalignment caused by night-shift work is associated with several age-related disorders (53). Adult, WT mice subjected to chronic jet-lag by shifting light-dark conditions by an 8-hour phase advance every 4 days had significantly shorter lifespans than control mice, increased levels of senescent immune cells, and increased inflammatory cell infiltration to the liver indicating chronic inflammation (54). Another jet-lag model revealed significantly shorter lifespans of aged (27-31 months old), but not young (8-12 weeks old) C57BL/6 male mice, suggesting circadian misalignment has more severe consequences in aged animals (55). The exact mechanisms responsible for premature ageing seen in human shift-workers are multifaceted and not fully understood.

A recent study found night-shift workers had increased plasma levels of C-reactive protein compared to day workers, indicating increased systemic inflammation (56). Importantly, night-workers had slightly decreased levels of long pentraxin 3 (PTX3), a pattern recognition receptor, which positively correlates with leukocyte telomere length, a marker of biological ageing. This suggests night-workers are more susceptible to premature ageing through increased systemic inflammation and loss of protective PTX3. Therefore, people who experience chronic circadian misalignment should consider the impact this may have on their health, and restoration of immune homeostasis may be a therapeutic target against age-related disorders in these people.

### Inflammation and Rhythmicity: A Reciprocal Relationship

Inflammation itself can directly affect circadian rhythmicity. TNFα inhibits the CLOCK/BMAL1-induced activation of E-box regulatory elements in clock-controlled genes in fibroblasts *in vitro*, and in livers of mice *in vivo* (5). Other recent studies in rheumatoid synovial cells have shown TNFα suppresses PER2, while inducing expression of BMAL1 by upregulating RORα (57). Additionally, long term treatment with IFN-γ reduced the amplitude of the circadian rhythm of Per1-luc expression in individual cultured rat SCN neurons (58), and LPS injection caused transient suppression of core clock genes in male rats *in vivo*. These studies highlight the complex, reciprocal relationship between inflammation and clock genes, and support the idea increased inflammation seen in older adults may result in dysregulation of the circadian rhythm.

## Future Directions and Conclusions

Circadian rhythms play an essential role in immune homeostasis and regulate the diurnal rhythmicity seen in leukocyte trafficking under both steady state and inflammatory conditions. Inflammation itself can inhibit clock gene expression, demonstrating a complex and reciprocal relationship between the two biological systems. In older adults, there is a parallel increase in systemic inflammation and dysregulated leukocyte trafficking, and also a reduction of circadian outputs, both of which can enhance the other, therefore increasing vulnerability to disease. Despite a multitude of research into circadian systems and leukocyte trafficking, there is a need for more research into chronotherapy to optimise timing of drugs and vaccine delivery in order to improve drug efficacy, reduce side effects, and target chronic inflammation, particularly in aged individuals. Similarly, current work on vaccination has focussed on either how responses to vaccines vary with increasing age, or on how responses vary with the time of administration, but not on the two angles combined. Circadian rhythm research can also be beneficial to the development of non-pharmacological treatment strategies. For example, diminished circadian output leads to reduced sleep quality in older adults, which subsequently dysregulates global circadian rhythmicity. Entrainment of peripheral clocks *via* regulating food intake and light exposure may help alleviate the effects of dampened circadian outputs seen in older adults, and help prevent one of the contributing factors for increased inflammation. Importantly, ageing research struggles to characterise changes that occur as a natural result of healthy ageing, versus those that are a condition of age-associated pathology, or changes in the circadian circuitry. More research into the circadian clock and inflammageing could determine if circadian rhythmicity can be used a sign of pathological ageing.

## Author Contributions

PN wrote the first draft of the manuscript. MC, JG, and GR contributed to manuscript revision, read and approved the submitted version.

## Funding

PN was supported by a Wellcome Trust PhD studentship (108871/Z/15/Z) and MC was supported by a Royal Society Dorothy Hodgkin Research fellowship (DH160044). JG is supported by the MRC (MR/S002715/1 and MR/P023576/1). GR is supported by the British Heart Foundation (FS/20/2/34799).

## Conflict of Interest

The authors declare that the research was conducted in the absence of any commercial or financial relationships that could be construed as a potential conflict of interest.
